# FEASIBILITY AND ACCEPTABILITY OF A LOVING-KINDNESS MEDITATION AMONG SOCIO-ECONOMICALLY DIVERSE OLDER ADULTS

**DOI:** 10.1093/geroni/igad104.3351

**Published:** 2023-12-21

**Authors:** Nirmala Lekhak, Tirth Bhatta, Carolyn Streb, Karenna Tran, Joel Snyder

**Affiliations:** University of Nevada, Las Vegas, Las Vegas, Nevada, United States; University of Nevada, Las Vegas, Las Vegas, Nevada, United States; University of Nevada, Las Vegas, Las Vegas, Nevada, United States; University of Nevada, Las Vegas, Las Vegas, Nevada, United States; University of Nevada, Las Vegas, Las Vegas, Nevada, United States

## Abstract

It has been established that contemplative practices such as meditation can aid in stress management and the prevention of mental health issues. However, whether one of these techniques, such as loving-kindness meditation (LKM), is feasible for promoting later-life self-compassion, reducing loneliness, and maintaining mental health is unclear. This study examined the feasibility and acceptability of a LKM intervention among socioeconomically diverse older adults. We employed a waiting list control design and mixed methods approach to randomize the participants (n = 59) into two groups. The same intervention was administered to the second group at a later time. We oriented and instructed the older adults on how to meditate and perform the LKM intervention. It contained phrases for the self, immediate family, others, and humanity. We gathered outcome data at baseline, post-intervention, four weeks following journaling, and four weeks without journaling. The intervention was feasible and acceptable (n = 54). One hundred percent of participants rated this meditation practice as either good or better. The range for acceptability was between 4 and 20, while the range for feasibility was between 3 and 15. The participants reported that they accepted (M = 16.8, SD = 2.9) and found this meditative practice feasible (M = 12.9, SD = 1.8). Participants who engaged in this intervention daily reported that this meditative practice helped them reduce anxiety and enhance self-awareness. Our findings highlight the need for large-scale contemplative interventions (including more music and silence) that can promote self-compassion and reduce loneliness in older adults.

